# PTPRO-mediated autophagy prevents hepatosteatosis and tumorigenesis

**DOI:** 10.18632/oncotarget.3353

**Published:** 2015-03-20

**Authors:** Wenjie Zhang, Jiajie Hou, Xiaochen Wang, Runqiu Jiang, Yin Yin, Jie Ji, Lei Deng, Xingxu Huang, Ke Wang, Beicheng Sun

**Affiliations:** ^1^ Liver Transplantation Center of the First Affiliated Hospital and State Key Laboratory of Reproductive Medicine, Nanjing Medical University, Nanjing, Jiangsu Province, P.R. China; ^2^ MOE Key Laboratory of Model Animal for Disease Study, Model Animal Research Center of Nanjing University, Nanjing, P.R. China

**Keywords:** PTPRO, autophagy, AKT, p53, nonalcoholic steatohepatitis

## Abstract

Autophagy plays a critical role in the progression of nonalcoholic steatohepatitis (NASH) and hepatocellular carcinoma (HCC). Protein tyrosine phosphatase receptor type O (PTPRO) was recently identified as a tumor suppressor, but little is known about its role in NASH. Here, we investigated the role of PTPRO-dependent autophagy in insulin resistance, lipid metabolism, and hepatocarcinogenesis. Wild-type (WT) and ptpro^−/−^ mice were fed a high-fat diet (HFD) for another 16 weeks after diethylnitrosamine (DEN) injection to induce NASH. Ptpro^−/−^ mice exhibited severe liver injury, insulin resistance, hepatosteatosis and autophagy deficiency compared with WT littermates. PTPRO deletion also promoted the induction of lipogenic target genes and decreases in β-oxidation-related genes. Increased activation of AKT and accumulation of cytoplasmic p53 was detected in ptpro^−/−^ mice, which in combination repressed autophagy. Intriguingly, hyperinsulinemia involving AKT activation was also exacerbated in HFD-fed mice due to PTPRO deletion. Activation of AKT induced stabilization of the MDMX/MDM2 heterocomplex, thus promoting p53 accumulation in the cytoplasm. Inhibition of AKT restored autophagy and p53 accumulation in hepatocytes, indicating that AKT acts upstream of p53. Due to hyperinsulinemia and autophagy deficiency, a HFD could aggravate steatohepatitis in ptpro^−/−^ mice. Importantly, the expression of PTPRO was much decreased in human steatohepatitis, which was associated with increased p62 accumulation. Together, these data indicate that PTPRO regulates insulin and lipid metabolism via the PI3K/Akt/MDM4/MDM2/P53 axis by affecting autophagy.

## INTRODUCTION

Nonalcoholic fatty liver disease (NAFLD), the hepatic consequence of metabolic syndrome, is the most common cause of chronic liver disease in both industrialized and developing nations [1–3]. NAFLD represents a continuum of disorders that range from simple steatosis to non-alcoholic steatohepatitis (NASH) to cirrhosis, which are generally considered irreversible and increase the prevalence of hepatocellular carcinoma [4, 5]. Thus NASH is predicted to become the leading indication for liver transplantation in the near future [6]. As an intermediate stage of NAFLD, although several hypotheses have been proposed, the mechanisms of NASH remain poorly understood.

Macroautophagy (hereafter referred to as autophagy) is an evolutionarily-conserved proteolytic process that delivers cell constituents to the lysosome forming the autophagolysosome for degradation [7]. Autophagy has been shown to not only play an important role in many critical biological processes during periods of starvation, but has also emerged as a primary quality control mechanism necessary for the maintenance of cellular homeostasis [8, 9]. Constitutive autophagy is also essential for preventing accumulation of misfolded/unfolded proteins and aged/damaged cellular organelles, such as mitochondria and endoplasmic reticulum (ER) [10, 11]. Defective autophagy has been shown to result in enhanced chromosomal instability, with failure to degrade damaged cellular components contributing to promotion of the development of a large number of diseases, including hepatocellular carcinoma [12]. Inhibition of autophagy obviously increases triglycerides (TGs) and lipid droplets (LDs) both *in vivo* and *in vitro* [13]. Recently, HFD-fed mice have been shown to exhibit defective hepatic autophagic function, which is reflected by decreases in the LC3-II/I ratio and increases in p62 expression [12].

Protein tyrosine phosphatase receptor type O (PTPRO), a receptor type of phosphotyrosine phosphatase (PTP), is an integral membrane protein which has been identified in many parenchymal cells (including lung, liver, and breast) [14, 15], whereas PTPROt (its truncated isoform) is expressed in osteoclasts, macrophages and B lymphocytes [16]. We have previously demonstrated that PTPRO represses development of hepatocellular carcinoma by down-regulation of STAT3 (signal transducers and activators of transcription 3). In contrast to the predominant pathways, PTPRO negatively regulates PI3K signaling [17], which is a key pathway involved in autophagy [18]. Autophagy is promoted by AMP-activated protein kinase (AMPK) and inhibited by mammalian target of rapamycin (mTOR) [19]. In other words, autophagy deficient animals exhibit increased mTOR and decreased AMPK activity. P53, as a tumor suppressor, has long been shown to play a pivotal role in regulating several aspects of the cell cycle, as well as apoptosis, metabolism, and oncogenic activation in mammalian cells [20]. Accumulating evidence suggests that cytoplasmic p53 can repress autophagy whereas nuclear p53 induces autophagy through transcription-dependent and –independent mechanisms [21–23]. However, the underlying mechanism remains elusive. Moreover, it is also unclear in NASH whether PTPRO promotes autophagy by controlling p53. To figure out the exact role of PTPRO in NASH and autophagy, we employed ptpro^−/−^ mice to conduct *in vivo* and *in vitro* experiments using NASH models and primary cells. A greater understanding of the potential effects of PTPRO on p53 may help to elucidate PTP functions in NASH.

## RESULTS

### PTPRO deletion in hepatocytes exacerbates steatosis and promotes tumorigenesis

**Figure 1:**
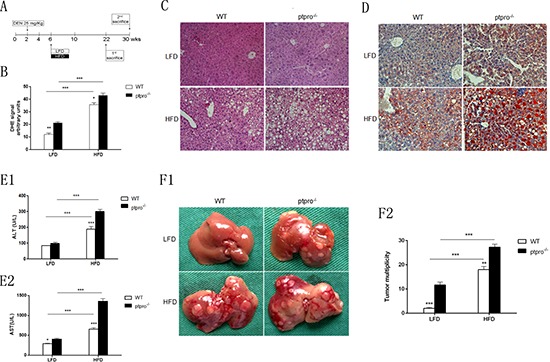
PTPRO deletion in hepatocytes exacerbates steatosis and promotes tumorigenesis **(A)** DEN was injected intraperitoneally (i.p.) into 14-day-old mice (WT and ptpro^−/−^). Four weeks later, the mice were fed a LFD or HFD for a further 16 weeks (*n* = 6 per group) or 24 weeks (*n* = 4 per group). **(B)** ROS levels were measured in LFD and HFD mouse specimens. **(C)** Liver sections of LFD and HFD mice were stained with H&E (original magnification, x200). **(D)** Liver sections of LFD and HFD mice were stained with Oil-Red O (original magnification, x200). **(E)** The levels of Serum aminotransferases were measured (*n* = 6 per group). **(F1)** Liver from 30-week-old DEN-treated mice. Tumorigenesis was investigated in WT and ptpro^−/−^ mice. **(F2)** Comparison of tumor multiplicity in male WT and ptpro^−/−^ mice (*n* = 4 per group). All data are expressed as mean ± SEM. *P < 0.05, **P < 0.01, ***P < 0.001.

To investigate whether PTPRO contributes to steatosis and tumorigenesis, we used a NASH-HCC animal model to monitor progression from obesity to HCC according to the method reported by Park [24]. Briefly, as shown in Figure 1A, on postnatal day 14 we injected DEN (25 mg/kg) into male mice and 4 weeks later fed them either normal chow (LFD) or a high-fat diet (HFD) until sacrifice (22 weeks). The other mice kept on feeding until the initiation of tumor formation. and then sacrificed (30 weeks). As expected, HFD-fed mice gained more weight compared to the LFD-fed mice and there was no difference between WT and ptpro^−/−^ animals (Supplementary Figure S1A). Since ROS normally increase during development of NASH, we examined ROS accumulation in WT and ptpro^−/−^ mice fed a LFD or HFD. Obesity significantly increased ROS accumulation (***P < 0.001). Additionally, in ptpro^−/−^ mice, more severe ROS production was induced (Figure 1B). Based on these phenotypes, H&E and oil red-O staining were employed to examine the degree of hepatic steatosis. Although ptpro^−/−^ mice did not show an obvious increase in hepatocellular ballooning (Figure 1C), it was able to promote fat droplet accumulation in LFD-fed groups (Figure 1D). In contrast, ptpro^−/−^-HFD-fed mice had significantly worse HFD-induced hepatic lipid accumulation. Moreover, significantly elevated serum ALT and AST activity indicated that severe hepatic injury had occurred in obese mice. Liver damage in ptpro^−/−^ mice was markedly more severe compared to WT mice (Figure 1E).

Because mice can develop obviously tumors at 8 months following DEN treatment, the livers of each group of mice were separated and tumor number and size were recorded after 30 weeks feeding. As shown in Figure 1F, WT mice fed a LFD exhibited much less and fewer tumor. On the other hand, mice fed a HFD presented larger tumor number and size, particularly in ptpro^−/−^ mice. These results indicate that ptpro^−/−^ in hepatocytes contributes to the development of steatosis and tumorigenesis in mice fed a HFD.

### PTPRO deletion is associated with lipid storage involved in lipogenesis and β-oxidation in NASH

**Figure 2:**
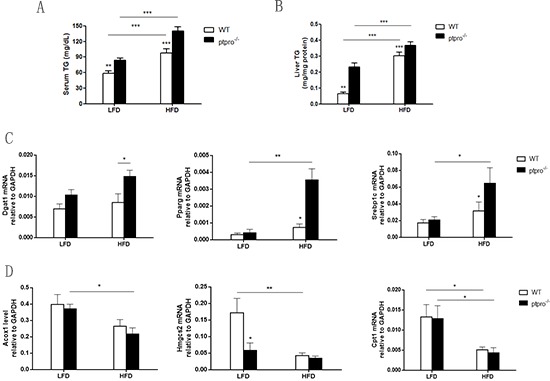
PTPRO deletion is associated with lipid storage involved in lipogenesis and β-oxidation in NASH **(A)** Serum triglycerides (TGs) were determined by a colorimetric assay (*n* = 6 per group). **(B)** Liver TGs were determined by a colorimetric assay (*n* = 6 per group). **(C)** Hepatic mRNA expression of lipogenesis-related genes (Dgat1, Pparg, Srebp1c) was determined by quantitative real-time PCR. The results are represented by ΔT values normalized to GAPDH. **(D)** Hepatic mRNA expression of β-oxidation related genes (Acox1, Hmgcs2, Cpt1) was determined by quantitative real-time PCR. The results are represented by ΔT values normalized to GAPDH. All data are expressed as mean ± SEM. *P < 0.05, **P < 0.01, ***P < 0.001.

Given the marked increase in lipid accumulation, we examined serum TG and liver TG in WT and ptpro^−/−^ mice fed a LFD or HFD. As illustrated in Figure 2A and 2B, both WT and ptpro^−/−^ mice exhibited elevated serum TG deposition, as well as liver TG. Of note, compared with WT mice, PTPRO deletion also resulted in elevated serum and liver TG. To further investigate the cause, we analyzed lipid storage gene expression involved in lipogenesis and β-oxidation in the liver by real-time PCR. Upon HFD feeding, the relative mRNA levels of lipogenesis-related genes, such as Dgat1, Pparg, and Srebp1c, were significantly enhanced (Figure 2C). Genes involved in β-oxidation including Acox1, Hmgcs2 and Cpt1 decreased in knockout, but not in WT mice (Figure 2D).

### Autophagy is suppressed in the livers of mice with hyperinsulinemia induced by PTPRO deletion in NASH

**Figure 3:**
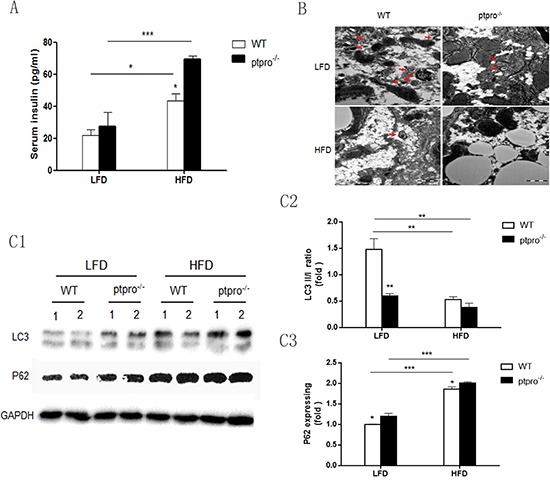
Autophagy is suppressed in the livers of mice with insulin resistance induced by PTPRO deletion in NASH **(A)** Serum insulin was determined by ELISA (*n* = 6 per group). **(B)** Mouse liver specimens prepared for transmission electron microscopy (TEM). Double membrane vesicles are indicated by red arrows (original magnification, x30K). **(C1–C3)** Levels of LC3I, LC3II and p62 in the liver were determined by immunoblotting. After densitometric analysis of blots corresponding to all samples, the results were quantitated using Image J software. All data are presented as mean ± SEM. *P < 0.05, **P < 0.01, ***P < 0.001.

Since hyperinsulinemia plays a pivotal role in the progression of NASH [25, 26], serum insulin was measured in our animal model. Elevated serum insulin was observed in HFD-fed mice, not only in WT animals but also in ptpro^−/−^ mice (Figure 3A). Hence, PTPRO deletion could contribute to hyperinsulinemia in mice. Moreover, we also observed reduced PTPRO expression and increased autophagy deficiency in animals fed a HFD (Supplementary Figure S1B).

Based on the serum results, we hypothesized that increased serum insulin correlates to autophagy status and leads to autophagy deficiency in the livers of mice. To test this hypothesis, we first employed TEM to observe autophagosomes (red arrows). As shown in Figure 3B, fewer autophagosomes were observed in HFD-fed mice, especially in ptpro^−/−^ mice. Atg8/LC3 is considered to be the most widely-used detector of autophagy. It can be divided into LC3-I and LC3-II according to whether or not lipidation occurs. A decrease in the LC3II/I ratio and p62 accumulation are the signs of impaired autophagy. Thus LC3 and p62 expression were examined in each group. Our results showed that obesity significantly reduced autophagy, furthermore, PTPRO deletion could aggravate it, as evidenced by a reduced LC3II/I ratio and increased p62 expression when analyzed by western blot (Figure 3C). Taken together, these observations confirm that PTPRO deletion promotes obesity-mediated hyperinsulinemia and autophagy deficiency in the liver.

### Regulation of proliferation and apoptosis by PTPRO in obesity

**Figure 4:**
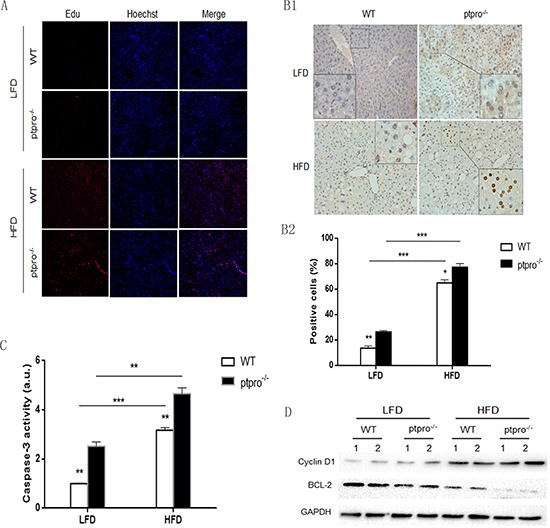
Regulation of proliferation and apoptosis by PTPRO in obesity **(A)** After 16 weeks of feeding with a LFD or HFD, mice were given Edu injections (5 mg/kg) one day before sacrifice. Liver sections were analyzed for hepatocyte proliferation based on BrdU (red) and Hoechst dye (blue). **(B1)** TUNEL staining for WT and ptpro^−/−^ liver. **(B2)** Apoptosis rates of mouse liver samples are shown as mean numbers of positive cells (*n* = 6 per group). **(C)** Caspase-3 activity in WT and ptpro^−/−^ mouse specimens after 16 weeks of feeding (*n* = 6 per group). The results are shown as absorbance units (AU). **(D)** Protein levels of Bcl-2 and CyclinD1 in WT and ptpro^−/−^ mouse specimens after 16 weeks of feeding detected by western blot. All data are presented as mean ± SEM. *P < 0.05, **P < 0.01, ***P < 0.001.

In order to evaluate liver cell proliferation in obesity, the proliferative ability of each group was evaluated by Edu cell proliferation assay. A HFD significantly enhanced the proliferative ability of liver cells, although PTPRO deletion caused no visible difference in proliferation (Figure 4A). Meanwhile, obese mice exhibited elevated CyclinD1 expression, but this was not observed in lean mice (Figure 4D). TUNEL (terminal deoxynucleotidyl transferase mediated dUTP nick end labeling) was also performed, as shown in Figure 4B1, and the result revealed that HFD-fed mice showed enhanced apoptosis compared with LFD-fed mice. Of interest, even though PTPRO deletion had no effect on proliferation, ptpro^−/−^ mice showed an increase in apoptosis. To confirm these results, we checked caspase-3 activity in the liver tissues of the different groups. As illustrated in Figure 4C, the results were similar to those obtained with TUNEL. Moreover, expression of the anti-apoptotic protein BCL-2 was downregulated in obese mice, especially in ptpro^−/−^ mice (Figure 4D). These results suggest that enhanced apoptosis may trigger compensatory proliferation of hepatocytes.

### Activation of AKT induces stabilization of the MDMX/MDM2 heterocomplex and accumulation of cytoplasmic p53 due to PTPRO deletion

**Figure 5:**
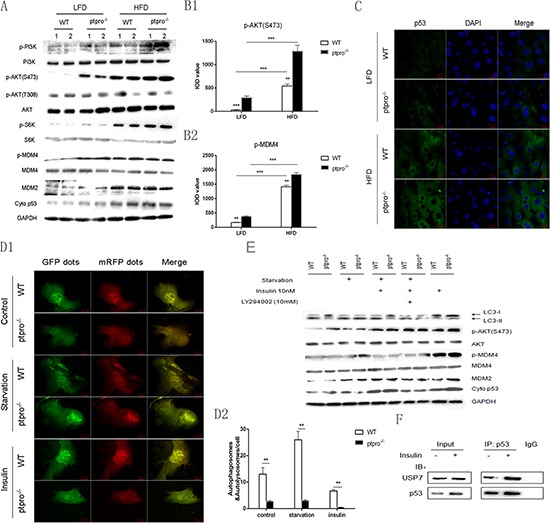
Activation of AKT induces stabilization of the MDMX/MDM2 heterocomplex and accumulation of cytoplasmic p53 due to PTPRO deletion **(A)** Mice were fed a LFD or HFD, then liver tissues were collected and the protein extracts were analyzed by western-blot for p-PI3K, PI3K, p-AKT, AKT, p-S6K, S6K, p-MDM4, MDM2 and cytoplasmic (cyto) p53. **(B)** Average integrated optical density (IOD) was obtained by analyzing five fields for each slide evaluated by Image-Pro Plus software (version 5.0) for IHC staining of p-AKT and p-MDM4. **(C)** After 16 weeks of feeding a LFD or HFD, liver frozen sections were used to detect subcellular localization of p53 (green) by immunofluorescence, cells were counterstained with 4′,6-Diamidino-2-phenylindole (DAPI) to visualize the nuclei. Scale bar, 10 μm. **(D1)** Primary hepatocytes were plated onto glass-bottomed culture chambers for 12 h, followed by transfection with mRFP-GFP-LC3 labeled adenovirus. Cells were then treated with EBSS or insulin as previously described. mRFP-GFP-LC3 was visualized by confocal microscopy. Scale bar, 20 μm. **(D2)** Quantification analysis of autophagy dots in hepatocytes. Data represent mean ± SEM from three independent experiments. **(E)** Hepatocytes were isolated from both WT and ptpro^−/−^ mice, the cells were treated with either DMEM or EBSS for 8 h, then insulin (10 nM) was added to the cell culture dish for 8 h. LY294002 (100 mM) was added to some of the cell cultures prior to insulin as noted. The protein levels of LC3-I and II, p-AKT, p-MDM4, MDM2 and cytoplasmic (cyto) p53 were detected by western blot. **(F)** Combination of p53 and USP7 in mouse hepatocytes treated with or without insulin, as detected by Co-IP. All data are expressed as mean ± SEM. *P < 0.05, **P < 0.01, ***P < 0.001.

Since it has been shown that the PI3K/Akt/mTOR/p70 ribosomal protein S6 kinase (p70S6K) pathway is a negative regulator of autophagy [27], we investigated this pathway using western blotting. As predicted, obesity induced upregulation of p-PI3K, p-AKT (S473) and mTORC2, all downstream targets of p-S6K expression. Furthermore, PTPRO deletion markedly promoted the phosphorylation of these proteins. In addition, we evaluated AKT (T308) phosphorylation, and observed no disparity between these groups (Figure 5A). Immunohistochemical staining of p-AKT (S473) and p-MDM4 was performed, and showed that p-AKT and p-MDM4 were increased in samples from obese mice, especially in ptpro^−/−^ mice (Figure 5B). Further, hepatocytes were isolated from both WT and ptpro^−/−^ mice and treated with insulin. As shown in Figure 5E, starvation was able to activate autophagy whereas treatment with insulin reversed this process. In addition, insulin-mediated autophagy deficiency was mostly prevented by the phosphatidylinositol 3-kinase inhibitor LY294002. Insulin treatment also induced AKT, MDM4 phosphorylation, as well as accumulation of MDM2 and cytosolic p53, which was largely prevented by LY294002 (Figure 5E). Similarly, in primary hepatocytes transfected with mRFP-GFP-LC3 adenovirus and monitored by confocal microscopy, nutrient starvation stimulated LC3-II aggregation while insulin inhibited the aggregation of puncta (Figure 5D). Of note, PTPRO markedly promoted autophagy in our culture system. These results demonstrate that PTPRO deletion promotes autophagy deficiency and that insulin signaling is responsible for the suppressed autophagy.

Published data indicate that cytoplasmic p53 is able to repress autophagy, so we assessed its cytoplasmic expression in primary cells and in mice by western blotting. As shown in Figure 5A and 5E, cytoplasmic p53 expression was increased in ptpro^−/−^ mice and cells. Moreover, immunofluorescence results confirmed our findings (Figure 5C). To explore the molecular mechanism responsible for p53 accumulation, as a substract of AKT kinase activity, phosphorylation of MDM4 at Ser367 was examined and was found to parallel AKT fluctuation. However, MDM4 lacks intrinsic E3 ligase activity. Given the findings that MDM4 is capable of forming stable heterodimers with MDM2 and stabilizing it, Mdm2 could induce nuclear export of p53 through mono-ubiquitination. MDM2 expression was also detected. Our results showed that MDM2 was also increased in ptpro^−/−^ mice (Figure 5A), indicating that MDM2 promotes p53 nuclear export by mono-ubiquitination. Furthermore, as shown in Figure 5F, we also observed a direct interaction between p53 and USP7 in primary hepatocytes with or without insulin treatment, which protects p53 from proteolysis.

Taken together, our data suggest that cytoplasmic accumulation of p53 by PTPRO is mediated by the PI3K/Akt/MDM4/MDM2 axis.

### Low expression of PTPRO in hepatocytes may contribute to the inhibition of autophagy and progression of NAFLD in human samples

**Figure 6:**
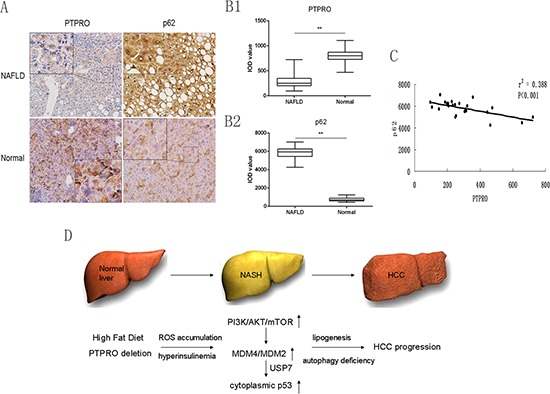
Low expression of PTPRO in hepatocytes may contribute to the inhibition of autophagy and progression of NAFLD in human samples **(A)** Selected images of IHC staining of PTPRO and p62 in both human NAFLD and normal liver tissues. **(B)** Average integrated optical density (IOD) was obtained by analyzing five fields for each slide evaluated by Image-Pro Plus software (version 5.0) (*n* = 24 for each group). **(C)** Correlation between expression of PTPRO and p62 in human NAFLD. **(D)** Schematic diagram of the involvement of PTPRO in the progression of NASH. PTPRO/PI3K/Akt/MDM4/MDM2/P53 signaling exacerbates autophagy deficiency, thus promoting hepatosteatosis and tumorigenesis. All data are expressed as mean ± SEM. *P < 0.05, **P < 0.01, ***P < 0.001.

To investigate the role of PTPRO in a clinical setting, 24 patients with benign liver conditions who had been diagnosed with NAFLD and were undergoing liver surgery were included in this study (Table 1). Immunohistochemical staining was employed to detect the expression of PTPRO and p62 and staining was quantified using image analysis software. As shown in Figure 6A and 6B, compared to normal tissues, the expression of p62 was significantly increased, whereas PTPRO was strikingly down-regulated in hepatocytes. We then analyzed the correlations between PTPRO and p62 expression in NAFLD specimens, and found that the expression of PTPRO was negatively correlated with that of p62 (Figure 6C, *r*^2^ = 0.388, ***P < 0.001). Therefore, our results suggest that low expression of PTPRO may contribute to the inhibition of autophagy and development of NAFLD.

**Table 1:** Patient characteristics

Clinical Features	NL *n* = 24	NAFLD *n* = 24
Age (years)	43.7 ± 8.4	47.2 ± 10.7
Gender		
Male	14	9
Female	10	15
Body mass index (kg/m2)	23.5 ± 2.9	29.4 ± 3.1^*^
Insulin (μU/l)	5.3 ± 2.5	14.6 ± 6.2^*^
Triglycerides (mg/dl)	98.8 ± 37.4	201.6 ± 54.9^*^
ALT (IU/l)	22.1 ± 6.7	74.1 ± 29.7^*^
AST (IU/l)	17.3 ± 3.5	56.5 ± 14.8^*^
γ-GT (IU/l)	31.2 ± 14.4	93.4 ± 53.2^*^
Steatosis (%)		
Grade 0	24 (100%)	
Grade 1		7 (29.2%)
Grade 2		11 (45.8%)
Grade 3		6 (25%)
Ballooning (%)		
Grade 0	24 (100%)	
Grade 1		6 (25.0%)
Grade 2		12 (50.0%)
Grade 3		6 (25.0%)
Lobular inflammation (%)		
Grade 0	24 (100%)	
Grade 1		7 (29.2%)
Grade 2		11 (45.8%)
Grade 3		6 (25%)

Abbreviations: ALT, alanine aminotransferase;

AST, aspartate aminotransferase;

γ-GT, gamma-glutamyltransferase;

NL, normal liver;

NAFLD, Nonalcoholic fatty liver disease;

Data are shown as mean ± S.D. or as number of cases (%).

* *p* < 0.05

## DISCUSSION

Autophagy is essential for maintaining homeostasis. If this important mechanism is interrupted, tumor growth is likely to occur. Recent evidence supports the hypothesis that during the early stages, autophagy restrains tumorigenesis. Since 2006, NASH has become the most rapidly-growing indication for HCC-related liver transplantation [6]. Both overweight and obesity significantly increase HCC risk [28, 29], but the underlying mechanisms by which they increase the risk of HCC and other cancers remain unclear. Emerging evidence has confirmed the observation that autophagy suppresses tumorigenesis but promotes growth of established tumors [11, 30]. As a precancerous lesion, the functions of autophagy in NASH remain elusive. Recent findings revealed defective autophagy in mouse and human NASH samples [12, 31]. However, some studies also detected elevated autophagic flux in the liver [32]. In our study, we used an HFD-induced model of HCC induction by DEN to monitor the obesity-HCC process. After 4 months of feeding a HFD, we demonstrated that defective autophagy in liver tissue could contribute to insulin resistance (IR), ROS production, and lipid-accumulation. Autophagy deficiency could promote the initiation of hepatic steatosis and progression of steatosis to HCC.

Our previous study demonstrated that the PTPRO level was significantly reduced in HCC compared to adjacent tissues [17]. Using mouse models, we confirmed that PTPRO expression was suppressed in mice fed a HFD compared to normal diet samples. A hallmark of impaired autophagy is the reduced LC3 II/I ratio and increased level of p62 [27, 33]. Our data indicate that PTPRO may regulate autophagy and lipid metabolism in the context of obesity and steatohepatitis. Ptpro^−/−^ mice showed decreased autophagy activity and elevated serum insulin. Insulin, TGs, and ROS can worsen liver function and induce liver inflammation, resulting in development of liver cancer [34]. Our results also show that PTPRO regulates lipid metabolism through the suppression of lipogenesis genes and the induction of β-oxidation-related genes. Furthermore, obesity significantly promoted tumorigenesis in the liver by the 7th month following DEN administration, especially in ptpro^−/−^ mice.

The class I phosphatidylinositol 3-phosphate kinase (PI3K)/Akt/mTOR/p70S6K pathway, as an upstream signaling pathway, negatively regulates autophagy [35]. It is associated with tumorigenesis and is critically altered in various types of tumor [36, 37]. Emerging evidence indicates that signaling cascades driven by AKT contribute to aberrant lipid accumulation and hepatocarcinogenesis [20, 38, 39]. Therefore, we examined this pathway using western blotting. Compared with WT-NASH mice, ptpro^−/−^ mice exhibited a significant increase in the levels of p-PI3K, p-AKT, and p-s6k, suggesting that activated Akt (S473) rather than AKT (T308) signaling results in autophagy deficiency and aberrant lipogenesis, which in combination promote hepatocarcinogenesis. Despite the lack of any obvious difference in proliferation between WT and ptpro^−/−^ mice, HFD-fed mice exhibited increased hepatocyte proliferation. Moreover, PTPRO deficiency significantly promoted cell apoptosis by reducing the action of the anti-apoptotic protein BCL-2 both in LFD and HFD groups, suggesting that compensatory hepatocyte proliferation is triggered by enhanced apoptosis, and AKT activation is likely to be responsible for enhanced cell proliferation. Enhanced liver damage gives rise to compensatory proliferation of initiated hepatocytes may explain augmented HCC development in obesity mice. Because hyperinsulinemia and IR are the crucial pathogenic signals for the development of NASH [26], in this study, we also found that insulin stimulation *in vitro* could lead to AKT, p-MDM2, MDM4 activation and impaired autophagy. The PI3K inhibitor LY294002 and AKT both obviously reverse this process.

P53, a well-known tumor suppressor, is normally mutated in human cancers [40]. Previous studies considered p53 to be a positive regulator of autophagy [41, 42]. However, recent studies challenged this notion [22, 43], since deletion, depletion or inhibition of p53 can trigger autophagy [43]. Tasdemir et al. and Morselli et al. both confirmed the observation that cytoplasmic p53 inhibits autophagy, whereas nuclear p53 promotes autophagy, which is involved in the AMP kinase and mTOR pathways [21, 23]. We demonstrated that ptpro^−/−^ mice exhibit a remarkable increase in p53 cytoplasmic expression through AKT/mTOR signaling *in vivo* and *in vitro*.

MDM4 (also known as HDMX and MDMX) and MDM2 are the main regulators of p53. Recent findings confirmed that AKT can promote stabilization of MDM4 via a post-transcriptional mechanism [44]. Along the same lines as Lopez-Pajares et al. [45], we also found that AKT can phosphorylate MDM4 at Ser^367^ that leads to stabilization of MDM4 and MDM2. Although MDM4 is structurally related to Mdm2, MDM4 lacks intrinsic E3 ligase activity. MDMX and MDM2 form stable heterodimers through their RING-finger domains [46]. As previously shown, low levels of Mdm2 activity induce mono-ubiquitination and nuclear export of p53, whereas high levels promote poly-ubiquitination of p53 and nuclear degradation [47, 48]. In our model, we observed elevated MDM2 and cytoplasmic accumulation of p53 in ptpro^−/−^ mice. We also show that USP7 physically interacts with p53, suggesting that the USP7-mediated de-ubiquitination of p53 inhibits its proteolysis. Accumulated p53 in the cytoplasm inhibits autophagy, leading to lipogenesis and hepatocarcinogenesis.

In summary, our observations underscore the importance of PTPRO in the progression of NASH. PTPRO is a negative regulator of autophagy via the PI3K/Akt/MDM4/MDM2/P53 axis. PTPRO deletion causes hyperinsulinemia and cytoplasmic p53 accumulation, which in turn promote hepatosteatosis and tumorigenesis.

## MATERIALS AND METHODS

### Animal model

Wild-type C57BL/6 mice were purchased from the animal center of Nanjing Medical University; PTPRO knockout C57BL/6 mice were donated by Dr. Bixby, University of Miami and were maintained and bred in the animal center of Nanjing Medical University. All mice were maintained in filter-topped cages and fed an autoclaved chow diet (low-fat diet, LFD; 12% fat) or high-fat diet (HFD; 60% fat; Research Diets).

All animals received care in compliance with the guidelines outlined in the Guide for the Care and Use of Laboratory Animals. In the DEN-induced HCC model, DEN (25 mg/kg) was injected intraperitoneally (i.p.) into 14-day-old male mice. After 4 weeks, mice were separated into two dietary groups and fed either a LFD or HFD until sacrificed.

### Human liver samples

Paraffin-embedded human liver tissues were acquired from benign liver condition (hepatic haemangioma) undergoing liver surgery with NAFLD at the First affiliated Hospital of Nanjing Medical University from January 2013 to May 2014 were involved in the study. Normal liver parenchymal tissues were obtained as controls from 24 patients with benign disease (hepatic haemangioma) without NAFLD. All liver tissues were read by a single hepatopathologist who was blinded to clinical data. Inclusion and exclusion criteria as described [49]. The degree of steatosis, hepatocellular ballooning and lobular inflammation were assessed as outlined by Kleiner et al [50] and Brunt [51]. Informed consent for gene expression analysis was obtained from each patient prior to surgery, and the study was approved by the institutional ethics committee in agreement with the Declaration of Helsinki.

### Hepatocyte isolation

Mouse livers were perfused *in situ* with 50 mL of Gibco liver perfusion medium (Invitrogen, 17701-038) followed by 45 mL of Gibco liver digestion medium (Invitrogen, 17703-034). The liver digests were filtered through a cell strainer and washed with Gey's balanced salt solution (Sigma, G9779) containing DNase I (2 mg/mL, Roche Diagnostics, 10104159001). The homogenate was centrifuged at 50 × g for 2 min at room temperature to collect the hepatocytes. Isolated hepatocytes were washed thoroughly with PBS. After determination of cell viability (95%), hepatocytes were plated into 6-cm dishes and maintained in DMEM (Sigma-Aldrich, D5546) containing 10% fetal calf serum with a combination of penicillin and streptomycin for further analysis.

### *In situ* detection of ROS

Liver ROS were detected in situ utilizing DHE, a dye that fluoresces upon reaction with ROS. Briefly, mouse liver specimens in each group were frozen in Tissue-Tek OCT and stored at −80°C. Frozen tissue was cut by cryostat into 5-μm-thick sections, which were mounted on Superfrost/Plus slides and stored at −80°C until analysis. For ROS detection studies, sections were incubated in PBS containing 2 nM DHE for 20 min at 37°C in a humidified incubator. The slides were then washed with room temperature PBS and analyzed by an epifluorescence microscope with excitation at 540 nm and emission at 605 nm.

### H&E, oil red O staining and immunohistochemistry

H&E staining was performed using a standard protocol for cryosections. Frozen tissue sections were stained with oil-red O (ORO) for lipid detection. For immunohistochemistry, all tissues were fixed in 4% paraformaldehyde overnight at 4°C, processed, sectioned to 5 μm slices, and dewaxed. Antigen retrieval was performed by heating the slides in the autoclave for 3 min using citrate buffer (pH 6.0). The slides were incubated with primary antibody (diluted at 1:50 to 1:400) overnight at 4°C. Negative controls were prepared by replacing the primary antibody either with serum or with antibody dilution buffer. The primary antibodies utilized were anti-PTPRO antibody (Sigma Aldrich, HPA034525), anti-AKT (S473) antibody (Bioworld, BS4007); the other antobodies utilized were the same as in Western blot analysis. Photographs were taken with the microscope (Nikon, ECLIPSE 50i) and software NIS-Elements v4.0, and average values of integrated optical density (IOD) were obtained by analyzing five random fields per slide using Image-Pro Plus software v. 5.0. Every index was detected a minimum of three times.

### Triglyceride and insulin measurement

Serum insulin was measured in mice after a 6 hrs food withdrawal with a commercially available Rat/Mouse insulin ELISA kit (Merck&Millipore, EZRMI-13K). Liver triglycerides were determined with colorimetric assay systems (Sigma-Aldrich) adapted for microtitre plate format. Liver TG was extracted in chloroform/methanol (2:1, v/v) and measured as above.

### Assay for serum transaminase activity

Serum samples from mice were obtained in each group. liver aminotransferases were used to assess the hepatocyte damage. ALT and AST were measured by chemical analyzer Hitachi 7600-10 in the Department of Clinical Laboratory in our hospital.

### Quantitative real-time PCR

Reverse transcription reactions were performed using the SuperScript First-Strand Synthesis System (Invitrogen, 11904-018). The RNA template was treated with DNase to avoid genomic DNA contamination. To determine the relative level of cDNA, real-time PCR analyses were performed using an Applied Biosystems 7300 Detection System (Applied Biosystems^®^, CA). The real-time PCR reaction was performed according to the protocol of the SYBR^®^ Premix Ex Taq™ kit (Takara, DRR041). Data were normalized with the GAPDH levels in the samples. The primers utilized for real-time PCR analysis were designed to span introns using primer analysis software (Oligo, version 6.54) (Supplementary Table S1).

### Adenovirus transduction and confocal microscopy

Primary hepatocytes were isolated from both WT and PTPRO mice liver, cells were plated onto glass bottom cell culture chamber (NEST, 801002). The next day, tandem mRFP-GFP-LC3 adenovirus (Hanbio) was introduced into cells. Some cells were then cultured in regular Dulbecco's modified Eagle's edium with 10% fetal bovine serum at 37°C. Cells were next pretreated with 10 nM insulin for 30 min, followed by incubation in the nutrient starvation medium Earle's balanced salt solution (EBSS) (Invitrogen, 14155063) or for 8 h, then fixed and observed with a confocal microscope (Leica TCS SP8). The images were manipulated with Zeiss LSM software.

### Transmission electron microscopy (TEM)

For EM, liver biopsies were fixed in suspension with 2.5% glutaraldehyde at 4°C overnight, and post-fixed for 1 h on ice with 1% osmium tetroxide. Then, tissues were dehydrated, infiltrated and embedded according to the usual methods. The ultrathin sections were stained for 20 min with 2% uranylacetate, followed by lead citrate and viewed in a Hitachi H-500 electron microscope (FEI, USA)

### Edu

Mice were injected i.p. with EdU (5 mg/kg) in PBS and mouse liver specimens were harvested at 24 h after injection. For staining fixed sections, pieces of the liver were formalin-fixed, embedded in paraffin, and sectioned. After paraffin removal, sections on glass slides were stained with 10 μM Alexa568-azide for 10–30 min, as described for fixed cells on coverslips except that the reaction and subsequent washes were performed in Coplin jars. Sections were counterstained with Hoechst and mounted for fluorescence microscopy.

### Terminal deoxynucleotidyl transferase mediated dUTP nick end labeling (TUNEL) assay

Formalin-fixed and paraffin embedded mouse liver specimens were sectioned into 5 μm slices, then deparaffinized in xylene and hydrated in graded ethanol. The TUNEL-positive cells were identified using a commercial kit (Roche, 11772465001) following the manufacturer's protocol. Photographs were taken with the microscope (Nikon, ECLIPSE 50i) and software NIS-Elements v4.0. Average numbers of TUNEL positive cells were recorded by counting five random fields per slide.

### Caspase-3 activity assay

Caspase-3 activity was checked in the liver tissues in each group. Protein samples (30 μg) were incubated with 200 μM of enzyme specific colorimetric caspase-3 substrate at 37°C for 2 h. Then the caspase-3 activity was detected by using Caspase-3 Activity Assay Kit (Calbiochem) and the absorbance unit (AU) was measured with a fluorometer at 405nm according to the manufacturer's instructions.

### Immunofluorescence

For immunofluorescence, fresh frozen sections were cut at 5-μm thickness using a cryostat. All tissues were fixed in fresh acetone followed by permeabilization in 0.2% Triton X-100/0.5% normal goat serum/PBS, then incubated with primary antibody against p53 (Cell signaling technology, #2527) and secondary antibodies labeled with FITC. After nuclear staining with the UltraCruz^™^ Mounting Medium (Santa Cruz, sc-24941), the tissues were visualized using Confocal Laser Scanning Microscope (Leica TCS SP8).

### Co-Immunoprecipitation (Co-IP)

Primary hepatocytes were isolated from C57BL/6 mouse as described above, and administrated with or without 6 hr of insulin treatment prior to protein extraction. Cytoplasmic protein were extracted by using Nuclear Cytoplasmic Extraction Reagents (Pierce, 78833), and a quantity of 30 μg protein extract was disposed as input. Immunoprecipitation was performed at 4°C overnight with 2 μg of anti-p53 antibody (abcam, ab26), and incubated with protein A agarose beads at room temperature for 2 hr (Santa Cruz, sc-2003). Then, the IPs and inputs were analyzed by sodium dodecyl sulfate-polyacrylamide gel electrophoresis, transferred to PVDF membrane, and immunoblotted with antibody against p53 or USP7 (Millipore, 2318812).

### Western-bolt analysis

Proteins were extracted from mouse tissues and cells using RIPA buffer containing fresh protease and phosphatase inhibitors, and quantified using a protein assay (Bio-Rad Laboratories, Hercules, CA). Protein samples (30 μg) were separated by SDS-PAGE and transferred to a nitrocellulose membrane. Immunoblotting was conducted using antibodies against PTPRO (Proteintech group, #12161), LC3 (Cell signaling technology, #12741), p62 (Cell signaling technology, #5114), PI3K (Cell signaling technology, #4249), p-PI3K(Y458/Y199) (Bioworld, BS4605), p-AKT(S473) (Cell signaling technology, #9271), p-AKT(T308) (Cell signaling technology, #9275), AKT (Cell signaling technology, #9272), p-s6k (Cell signaling technology, #9205), s6k (Cell signaling technology, #9202), p-MDM4(S367) (Abcam, ab122926), MDM4 (Abcam, ab76362), MDM2 (Abcam, ab3110), p53 (Cell signaling technology, #2527), Cyclin D1 (Santa Cruz, sc-20044), Bcl-2 (Santa Cruz, sc-130307). The results were visualized using a chemiluminescent detection system (Pierce ECL substrate western blot detection system, Thermo Scientific, 32106) and exposure to autoradiography film (Kodak XAR film).

### Statistical analysis

Results are expressed as mean ± SEM. Comparisons between the two groups were performed using the unpaired Student's *t* test. Correlations between parameters were determined using Pearson correlation and linear regression analysis, as appropriate. All statistical analyses were performed using SPSS statistical software version 13.0, and two-tailed tests were applied to all data unless otherwise specified; a *p*-value < 0.05 was considered to indicate a statistically significant result.

## SUPPLEMENTARY FIGURES AND TABLE



## Abbreviations

PTPRO: Protein tyrosine phosphatase receptor type O
NASH: nonalcoholic steatohepatitis
HCC: hepatocellular carcinoma
LFD: low fat diet
HFD: high fat diet
DEN: diethylnitrosamine
NAFLD: Nonalcoholic fatty liver disease
ER: endoplasmic reticulum
TG: triglyceride
LDs: lipid droplets
STAT3: signal transducers and activators of transcription 3
AMPK: AMP activated protein kinase
TEM: transmission electron microscopy
ORO: oil-red O
HE: Hematoxylin and eosin
qRT-PCR: quantitative reverse transcription-polymerase chain reaction
EBSS: Earle's balanced salt solution
TUNEL: Terminal deoxynucleotidyl transferase mediated dUTP nick end labeling
Co-IP: Co-Immunoprecipitation
IR: insulin resistance
