# Supplementary material for: PTPRO-mediated autophagy prevents hepatosteatosis and tumorigenesis
Source: Oncotarget. 2015 Mar 20;6(11):9420–33. doi: 10.18632/oncotarget.3353 (PMC4496227; doi:10.18632/oncotarget.3353)
Supplement: Supplementary file 1 [file oncotarget-06-9420-s001.pdf]

## SUPPLEMENTARY FIGURES AND TABLE

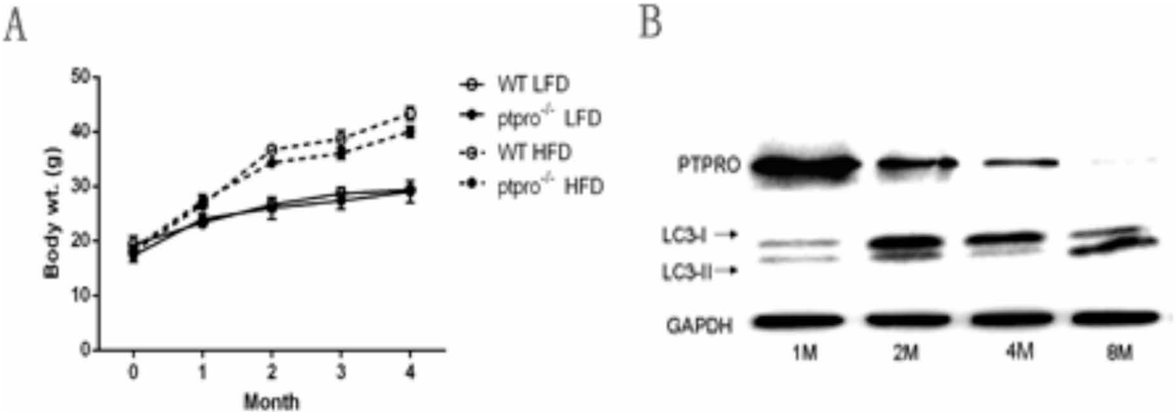


**Supplementary Figure S1: (A)** Body weight gain during NASH development in WT and ptpro-/- male mice kept on LFD or HFD after DEN administration (n = 6 per group). **(B)** Mice were feeding HFD, then liver tissues were collected at 1M (month), 2M, 4M, 8M and the protein levels of PTPRO, LC3I/II were analysis by Western-blot.

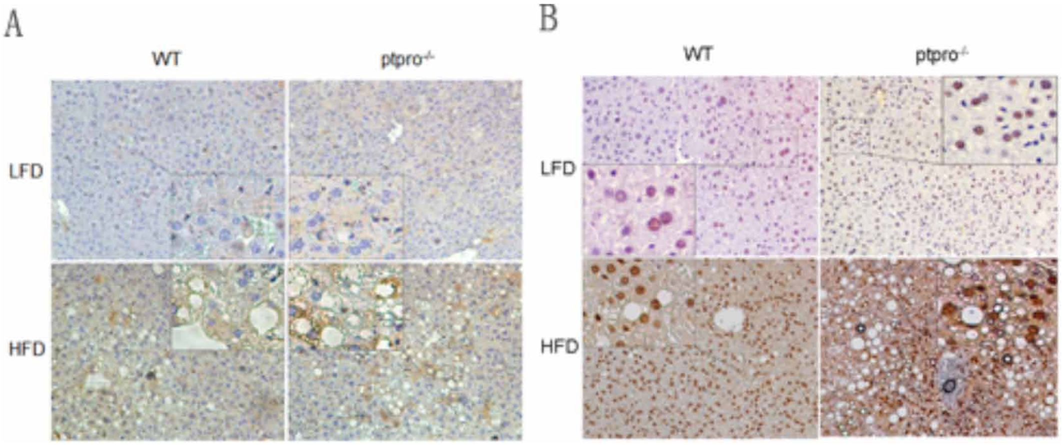


**Supplementary Figure S2: (A)** Representative figures for IHC staining of p-AKT (S473) in LFD or HFD treated liver of WT and ptpro$^{-/-}$ mice. **(B)** Representative figures for IHC staining of p-MDM4 in LFD or HFD treated liver of WT and ptpro$^{-/-}$ mice.

### Supplementary Table S1: Primer sequences for amplification of target genes

| Gene | Forward | Reverse |
|---|---|---|
| Dgat1 | CTGATCCTGAGTAATGCAAGGTT | TGGATGCAATAATCACGCATGG |
| Pparg | TCTTCCATCACGGAGAGGTC | GATGCACTGCCTATGAGCAC |
| Srebp1c | CTTTGGCCTCGCTTTTCGG | TGGGTCCAATTAGAGCCATCTC |
| Acox1 | TAACTTCCTCACTCGAAGCCA | AGTTCCATGACCCATCTCTGTC |
| Hmgcs2 | GAAGAGAGCGATGCAGGAAAC | GTCCACATATTGGGCTGGAAA |
| Cpt1 | AGTGGCCTCACAGACTCCA | GCCCATGTTGTACAGCTTCC |
| Gapdh | GCTGGTCATCAACGGGAAA | ACGCCAGTAGACTCCACGACA |
